# Hand Selection in Dribbling Phases: An Analysis of Non-Dominant Hand Usage and Dribble Change in Basketball

**DOI:** 10.3390/sports11110226

**Published:** 2023-11-14

**Authors:** Keisuke Onodera, Masaki Takeda

**Affiliations:** 1 Faculty of Education and Welfare, Biwako-Gakuin University, 29 Fuse-cho, Higashiomi 527-8533, Shiga, Japan; 2 Graduate School of Health and Sports Science, Doshisha University, 1-3 Tatara, Miyakodani, Kyotanabe 610-0394, Kyoto, Japan; 3 Faculty of Health and Sports Science, Doshisha University, 1-3 Tatara, Miyakodani, Kyotanabe 610-0394, Kyoto, Japan; mtakeda@mail.doshisha.ac.jp

**Keywords:** basketball dribbling, non-dominant hand, hand preference, hand selection, game situations, dribbling skills

## Abstract

This study investigates the influence of different dribbling phases on hand selection among basketball players across various categories. A total of 33 guard players, including 11 from high school, college, and NBA teams each, were observed. Video data analysis was utilized to determine the frequency of players using their dominant hands (DHs) and non-dominant hands (NDHs) during in-game dribbling phases. The dribbling phases were classified into three categories: First (the initiation of the dribble), Middle (during the dribble but not in First and Last), and Last (the completion of the dribble). Percentage, means, and standard deviations were computed for each category within the First, Middle, and Last measurements. A two-factor analysis of variance (ANOVA) was conducted, considering player category and dribbling phase as factors. The ANOVA revealed significant main effects of player category (*p* < 0.01) and dribbling phase (*p* < 0.01). Post hoc multiple comparisons using Holm’s method indicated that, in the First phase, players exhibited a 6.5% higher preference for using their NDHs (43.4 ± 11.9%) compared to the Middle phase (36.9 ± 13.9%) (*p* < 0.05). Similarly, in the Last phase, players displayed a 5.3% greater inclination towards using their NDHs (42.2 ± 11.7%) compared to the Middle phase (*p* < 0.05). These findings provide quantitative evidence that the specific dribbling phase influences hand selection during gameplay. The implications of these results are significant for basketball coaches, as they can design targeted training programs and drills that simulate game scenarios and encourage NDH usage. By understanding the factors influencing hand choice, players can enhance their versatility and adaptability on the court. Furthermore, these findings contribute to player performance, skill development, and strategic decision making in dribbling phases.

## 1. Introduction

Ball control holds significant importance for basketball players as it entails the ability to dribble the ball while changing directions, ultimately contributing to successful performances [1,2]. To dribble in basketball, many studies (e.g., [3,4,5]) indicate the need to control the ball with both the non-dominant hand (NDH) and the dominant hand (DH) to protect the ball from defenders. However, the factors that influence the use of the NDH in basketball are not yet fully understood [6].

Previous research indicates that factors such as skill level and the amount of practice influence NDH usage in basketball. For example, Stöckel and Weigelt [7] investigated the plasticity of handedness in basketball players and found that expert players exhibited decreased one-hand bias and inter-manual performance asymmetry, indicating increased proficiency with their NDHs. The authors suggest that the expert players’ experience in a highly complex and dynamic sport environment leads to a more balanced use of both hands. Gualdi et al. [8] conclude that these changes are due to training volume.

Several studies have examined other factors affecting the usage of NDHs in basketball. Giovanini et al. [6] investigated the influence of pressure on the use of the NDH in basketball. The results show that the frequency of NDH use for dribbling did not differ significantly between high- and low-pressure game conditions, such as during the National Basketball Association (NBA) Finals compared to the regular season, while players tend to use their NDHs less frequently for passing when facing high-pressure game conditions. Another study by Esteves et al. [9] investigated the influence of a defender’s body orientation on the directional preferences of experienced basketball players during dribble penetration in a laboratory setting. They found that players tended to use their DHs more when the defender was oriented towards their non-dominant side, suggesting that the NDH use would be influenced by the posture of defender in non-game settings. Limited research exists on how specific factors, especially across different player categories, impact hand selection during actual games. Moreover, the present literature barely explores the influences beyond a player’s skill level on the selection of hands during gameplay.

According to Glazier et al. [10], our movements are constrained by the interactions between the individual, the environment, and the task. Stöckel and Weigelt [7] noted that the characteristics of the individual, including skill level and practice, influence hand choice in basketball. However, the effects of the environment and task on hand choice in game settings warrant further investigation. In the studies by Stöckel and Weigelt [7] and Giovanini et al. [6], dribbling was analyzed by focusing only on the percentage of NDHs in the total number of dribbling controls performed in a game, and the overall trend was quantified and analyzed. This study, based on the FIBA Official Basketball Rules [11], categorized dribbling into three phases: ‘First’, ‘Middle’, and ‘Last’. According to these rules, a dribble begins when a player, in control of a live ball, bounces it and touches it again before another player does. It concludes either when the ball is touched with both hands at once or when allowed to rest in the hand(s) (Rule24.1.2). We hypothesize variations in hand usage across these phases. Regarding the First phase, Krause and Nelson [12] stated that “the critical cue for the live-ball offensive player is to attack the front or forward foot of the defender.” Additionally, Esteves et al. [9] concluded that the defender’s body orientation affects the way penetration occurs in a laboratory setting. Therefore, we hypothesized that the ratio of the First phase would differ from the Middle phase in actual games. The ‘Last’ phase refers to the hand that was used for the final dribble before players shot or passed the ball, or pivoted. Yashvant [13] pointed out that “when you start dribbling, you must dribble constantly as you move, until you pass, shoot, or stop dribbling to plant on your pivot foot.” Krause and Nelson [12] stated that “players in possession of the ball should avoid dead-ball situations whenever possible unless a pass or shot is anticipated.” Considering these statements, this analysis determines which hand was used to control the dribble when the decision was made to move towards passing, shooting, pivoting, etc. Therefore, we expected the Last phase to be more influenced by the environment and the task [10] than the overall dribbling trend, leading us to hypothesize that the ratio of the Last phase would differ from the Middle phase in actual games. As a separate analysis of dribbling phases, we analyzed Dribble Change (DC), which refers to the act of switching the controlled hand while dribbling. Although DC is an important skill in basketball and numerous studies have been conducted on this topic (e.g., [14,15,16]), we did not find any study focusing on the NDH–DH relationship. DCs are executed to avoid losing the ball or gain an advantage over the defender. Therefore, we hypothesized that the environment and the task [10], such as defensive pressure, player positioning, and court awareness, would influence hand choice during DCs, with no one-side bias. In bridging the gap between the nuances of dribbling phases and diverse player categories, our research offers a pioneering perspective that remains largely untouched in the existing studies. Our insights not only deepen the academic understanding of hand selection in basketball but also present actionable takeaways for training and coaching. By shedding light on these specificities, we aspire to enhance on-court decision making and performance. Thus, this investigation stands as a seminal contribution to both scholarly discussions on basketball techniques and pragmatic advancements in the sport’s training methodologies.

Therefore, this study aimed to investigate how different dribbling phases (i.e., First, Middle, Last, and Dribble Change) affect the hand choice of players from different categories (i.e., high school players, college players, and NBA players) during basketball games, in addition to analyzing the percentage of the NDH usage in the overall trend of dribbles. We classified players into three categories: high school players, who are emerging talents and still honing their skills; college players, who possess more refined skills and are exposed to higher competitive levels; and NBA players, who are professionals at the pinnacle of the sport. We hypothesized that the frequency of use would vary depending on the dribbling phase. By further exploring these factors, coaches and trainers can develop training programs that are specifically designed to improve NDH dribbling skills in basketball players, which can ultimately lead to better on-court performance. This study seeks to fill the knowledge gaps related to the topic being discussed, providing insights to enhance training methods and deepen our understanding of hand selection in basketball.

## 2. Materials and Methods

### 2.1. Definition of Dribble

The dribble is defined based on FIBA basketball rules. According to the FIBA Official Basketball Rules [11], “A dribble is the movement of a live ball caused by a player in control of that ball who throws, taps, rolls or bounces the ball on the court (Rule24.1.1). A dribble starts when a player, having gained control of a live ball on the court throws, taps, rolls or bounces it on the court and touches it again before it touches another player. A dribble ends when the player touches the ball with both hands simultaneously or permits the ball to come to rest in one or both hands (Rule24.1.2)”.

### 2.2. Subject of the Study

A total of 33 players were selected for this study, 11 each from the first guard position at different levels, including high school players, college players, and NBA players. Players were selected based on specific criteria relevant to their level of play. For the male high school players, starting individuals were chosen from teams that had progressed to the top 16 of a specific prefectural tournament in Japan, with ages ranging from 15 to 18 years. Similarly, starting male college players, aged between 18 and 22, were affiliated with Division 1 teams in Japan. NBA participants in this study were also starting point guards for their respective teams. The guard position was chosen because guards are known for their frequent use of dribbling as an essential skill for playmaking. Previous research has demonstrated that guards often need to acquire more advanced dribbling skills compared to players in other positions [1,17]. Furthermore, Gualdi et al. [8] state that the guard position requires the use of both hands more than other positions. Therefore, analyzing the dribbling performance of guards can provide valuable insights into the importance of dribbling in basketball. We chose 33 participants to ensure a balanced representation across all categories. The number was derived from an a priori power analysis using G*Power, targeting a sample size between 30 and 36 participants for effective analysis. Considering our data availability and aim for a balanced representation across high school, college, and NBA categories, we chose 11 participants from each, totaling 33. This sample size aligns with the range suggested by our power analysis.

The data was collected from the videos of 33 games of each player, including recorded videos of high school players and uploaded videos from the internet of college players and NBA players. Players were identified as first guards based on the team list, in-game commentary, and author identification, especially for college and NBA players, where videos were primarily sourced from edited game broadcasts. It is imperative to emphasize that while these videos were not specifically recorded for research purposes, they represent the most comprehensive and accessible data sources available. This approach aligns with the methodologies adopted in previous studies, such as those by Stöckel and Weigelt [7] and Giovanini et al. [6], where edited video footage was used, highlighting the standard methodologies being used. Furthermore, the DH and NDH of players were identified based on the hand which they used for the free throw and based on a previous investigation [6,7]. Out of the total 33 players, one player from college and two players from the NBA were left-handed, while the remaining 30 players were right-handed. In terms of their on-court contributions, high school players had an average playing time of 26.2 ± 6.3 min, scoring 8.5 ± 4.1 points, having 1.5 ± 1.0 assists, and 1.4 ± 1.4 turnovers per game. College players, with an average playtime of 27.5 ± 9.0 min, recorded 8.1 ± 6.9 points, 2.0 ± 1.0 assists, and 1.4 ± 1.2 turnovers. NBA players, playing for an average of 30.2 ± 6.8 min, scored an impressive 14.5 ± 9.5 points, had 5.9 ± 5.2 assists, and 1.3 ± 1.4 turnovers.

### 2.3. Measurements

In this study, we measured the number of times the subject players controlled the ball with their DHs and NDHs during their dribbles throughout their playing time in a game in which they participated. All dribbles that could be measured on video were included in this study. Initially, as detailed in Section 2.3.1, we analyzed the dribbling phase by segmenting it into three categories: First, Middle, and Last. Secondly, dribble changes were included in the analysis of dribble phases, as shown in Section 2.3.2. Since there might be expected differences in the number of times the ball is touched for dribbling between players and categories, and since this study focuses on the ratio of NDH use, we calculated the ratio and analyzed the percentages from the measured values (number of times) for each player. 

#### 2.3.1. Ratio of First, Middle, and Last Dribbles Controlled with NDHs

In the analysis of dribbling phases, we measured whether the DH or NDH was used to start the dribbling (First phase) in the playing time. We also assessed the number of dribbles controlled with either the DH or NDH that did not fall under the categories of “First” or “Last” phases, and this phase was termed as the “Middle” phase. Lastly, we measured whether the DH or NDH was used just before completion of the dribble to pass, shoot, pivot, etc. (Last phase). However, turnovers that occurred before the completion of the dribble were excluded from the Last category for analysis purposes. Following the measurements, the NDH ratios for the First, Middle, and Last phases were calculated for each player.

#### 2.3.2. Ratio of Dribble Changes from NDHs to DHs (DCs)

As a separate analysis of dribbling phases, we measured the number of times players changed the controlling hand while dribbling from their DHs to NDHs and from NDHs to DHs. To analyze one-side bias in the DC phase, we calculated the rate of DCs from NDHs to DHs for each player.

#### 2.3.3. General Description of the Data Sample

The measurements were performed by the sole author. In order to verify the standardized subjective evaluation in this research, one game was extracted and a collaborator with professional basketball experience was asked to perform the measurement. This was then compared with the measurements obtained by the researcher in this study. The agreement rates were: First phase at 100%, Middle phase at 99.6%, Last phase at 98.6%, and DC at 98.1%.

Due to video editing and camera limitations, it was not possible to capture all dribbles of college players and NBA players, as some dribbles may have occurred while the game was in progress and when the camera was focused on other players. Therefore, all dribbles that could be measured were included in this study. Although there may have been some recording inaccuracies in the recorded values, it is believed that the data were adequate for analysis, as this study was designed to analyze the ratio of DH and NDH dribbles used, rather than the absolute number of dribbles. Furthermore, as detailed below, it is believed that we were able to obtain a sufficient number of dribble sequences across all categories to accurately compute the ratios. 

For each category, per player, the male high school players had 157.0 ± 70.0 dribble contacts, college players had 158.6 ± 69.1, and NBA players had 261.0 ± 126.2. During the measurements, a series of dribbles (from catching the ball and initiating the dribble to its completion) were counted and equaled 44.1 ± 16.1 times for high school players, 39.8 ± 11.1 for college players, and 50.5 ± 14.5 for NBA players. Among the measured series of dribbles, there were instances where it was not possible to determine the hand with which the dribble was started (First phase) or ended (Last phase). Specifically, for the NBA players, there were 3.6 ± 1.4 of these instances, and for the college players, there were 3.4 ± 1.3 instances where the ‘First’ phases could not be determined. In terms of the Last phase, there were 0.4 ± 0.5 instances for NBA players and 0.3 ± 0.5 instances for college players where it was not possible to determine the hand which initiated or finished a dribble.

### 2.4. Statistical Analyses

After completing the measurements, we calculated the percentages, means, and standard deviations for each category for the First, Middle, and Last phase measurements, as described above. All results are presented as means ± standard deviation. When conducting ANOVA, normality was checked using the Shapiro–Wilk test and homogeneity of variance was verified with the Levene test. Both tests met their assumptions. To examine whether dribbling phases influenced hand selection and whether there were differences between categories, we conducted a two-factor analysis of variance (ANOVA) with three levels for each factor: dribbling phase (First, Middle, Last) and category (high school players, college players, and NBA players). Main effects were analyzed using a 3 × 3 two-factor ANOVA; and, if the main effects were significant, multiple comparisons were performed using the Holm method.

For the measurement of the ratios of DCs from NDHs to DHs, a one-factor ANOVA was used to examine effects across categories. In addition, we conducted a one-sample *t*-test to assess whether the percentage of the DC ratios of NDHs to DHs for each category significantly differed from a baseline value of 50. This particular baseline was selected as it symbolizes a neutral scenario: where players exhibit no discernible preference for either hand, suggesting an equivalent propensity to utilize the DH or NDH during DCs. The data were analyzed using IBM SPSS (version 26.0, IBM, Armonk, NY, USA). Statistical significance was set at 5%. 

## 3. Results

### 3.1. Two-Factor ANOVA (Dribbling Phases × Category)

**Figure 1:**
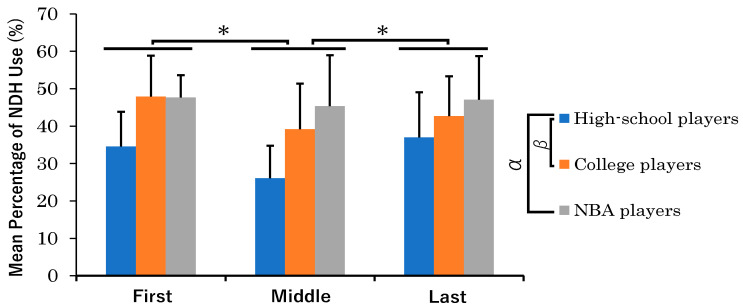
Mean percentage of NDH use by category (high school players, college players, and NBA players) in each the dribbling phase (First, Middle, Last). Error bars indicate standard deviations within each group. There were significant main effects observed for both category and dribbling phase. In post hoc multiple comparisons for category, α indicates *p* < 0.01 and β denotes *p* < 0.05. An asterisk (*) signifies *p* < 0.05 for the dribbling phase. The interaction effect between category and dribbling phase was not significant.

The two-factor ANOVA was conducted to understand the interaction between different phases of dribbling and player categories in terms of NDH usage. The results of the two-factor ANOVA for the percentage of dribbles made with the NDHs are shown in Figure 1. For the First, Middle and Last phases in the dribbling phases, the percentages of dribbles made with the NDHs were as follows: For the First phase, 34.6 ± 9.2% for high school players, 47.9 ± 11.0% for college players, and 47.6 ± 6.0% for NBA players; for the Middle phase, 26.1 ± 8.7% for high school players, 39.2 ± 12.3% for college players, and 45.4 ± 13.6% for NBA players; for the Last phase, 37.0 ± 12.0% for high school players, 42.7 ± 10.7% for college players, and 47.1 ± 11.6% for NBA players. 

The two-factor ANOVA revealed a significant main effect of category (F [2,30] = 7.34, *p* = 0.003) and a significant main effect of dribbling phase (F [2,30] = 5.69, *p* = 0.007). However, the interaction effect between category and dribbling phase was not significant (F [4,60] = 1.37, *p* = 0.260).

To further delineate specific differences among player categories regarding their NDH use, we applied post hoc multiple comparisons using Holm’s method. Post hoc multiple comparisons for category revealed a significant difference between high school and college players (t [30] = −2.77, padj = 0.019), with college players (43.2 ± 12.2%) using their NDHs significantly more often than high school players (32.6 ± 10.1%). NBA players (46.7 ± 11.5%) were also significantly more likely to use their NDHs than high school players (t [30] = 3.67, padj = 0.003). However, there was no significant difference between college players and NBA players (t [30] = −0.90, n.s.).

To discern the differences across dribbling phases, post hoc multiple comparisons were applied using Holm’s method. Post hoc multiple comparisons for dribbling phases revealed significant differences between the First and Middle phases (t [30] = −2.74, padj = 0.021), with players using their NDHs significantly more often in the First phase (43.4 ± 11.9%) than the Middle phase (36.9 ± 13.9%). In the Last phase (42.2 ± 11.7%), players were also significantly more likely to use their NDHs than in the Middle phase (t [30] = 2.99, padj = 0.017). However, there was no significant difference between the First and Last phases (t [30] = 0.57, n.s.).

### 3.2. One-Factor ANOVA and One-Sample *t*-Test of the DC Ratios from NDHs to DHs

**Figure 2:**
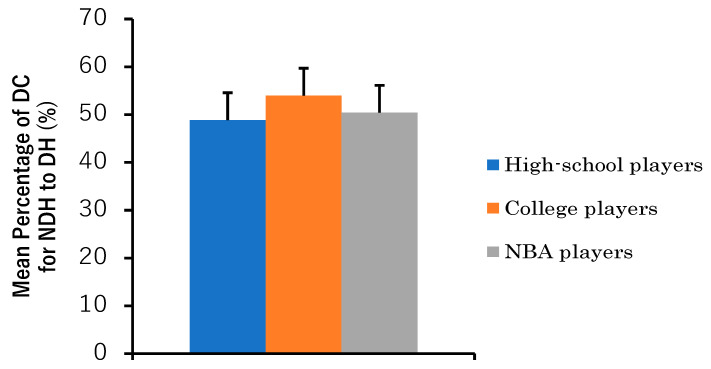
Mean percentages of DCs for NDHs to DHs by category (high school players, college players, and NBA players). Error bars indicate standard deviations within each group; 100× total number of DH changes from NDH to DH/(total number of DCs from DH to NDH + total number of DCs from NDH to DH) was used to calculate the values per player and the means and standard deviations per category. No main effect was found with the one-way ANOVA. One-sample *t*-tests were used to compare the means of each category with a reference value of 50, and no significant differences were found in any of the categories.

We conducted a one-factor ANOVA and a one-sample *t*-test to discern if there were any notable differences in the propensity of players to DCs from NDHs to DHs across player categories. The results of the one-factor ANOVA and one-sample t-test for the percentages of the DCs from NDHs to DHs are presented in Figure 2. The percentages of the DCs from NDHs to DHs for high school players, college players, and NBA players were 48.8 ± 6.7%, 54.0 ± 12.7%, and 50.4 ± 5.6%, respectively.

The one-factor ANOVA showed that the main effect of category was not significant (F [2,30] = 0.89, *p* = 0.426). Furthermore, one-sample *t*-tests showed no significant differences from the baseline value of 50 for all categories: high school players (t [10] = −0.55, *p* = 0.595), college players (t [10] = 0.99, *p* = 0.345), and NBA players (t [10] = 0.22, *p* = 0.834).

## 4. Discussion

### 4.1. Differences by Category

Our results showed that NBA players and college players used their NDHs significantly more often than high school players. Due to the different subject matter from the study by Stöckel and Weigelt [7], there were natural differences in the numerical values of the measurements, and the results of multiple comparisons also showed no significant differences between college players and NBA players. However, these findings are largely consistent with the results of Stöckel and Weigelt [7], who reported a significant difference in NDH use between professional, semi-professional, and amateur players. Considering the dynamic interplay highlighted by Glazier and Davids [10], where individual attributes, environmental constraints, and task demands come together to shape movement behaviors, it can be inferred that ‘Category’ likely reflects individual factors. This perspective underscores the notion that a player’s skill level, an intrinsic individual attribute, influences hand choice during dribbling in the context of specific environmental and task conditions. Stöckel and Weigelt [7] concluded that players in higher categories dribbled more with their NDHs because they practiced more with both their DHs and NDHs. Furthermore, Gualdi et al. [8] state that training volume is an important factor that enables proficient use of both hands in a game. Further research is needed to clarify how much the use of the NDH increases as skill level improves through practice. However, it can be concluded that the results of this study support previous studies that have shown that players with higher skill levels generally use their NDHs more when dribbling phases are not considered.

### 4.2. Effects of Dribbling Phases on Hand Selection

In assessing hand selection during dribbling, we hypothesized that the frequency of use would vary depending on the specific dribbling situation. Consistent with this, our study found that players used their NDHs significantly more often in the ‘First’ and ‘Last’ phases compared to the ‘Middle’ phase. Among the different phases, the First and Last are the ones in which the tendency to use one side more than the other side is not recognized, or at least less than Middle phase. The reason for why this occurs needs further research, but one possibility is that defensive pressure from the opponent effects this tendency. As mentioned above, with respect to the First phase, Krause and Nelson [12] provide recommended directions for each defensive orientation. Esteves et al. [9] also found that in practical one-on-one situations, the defender’s body orientation influenced the direction of the dribble, regardless of the level of basketball. This study demonstrates the influence of dribbling phases on hand selection during gameplay. Additionally, in previous studies [12,13], it was recommended to continue dribbling until the next play, such as a pass or shot, can be predicted. Therefore, the Last phase, which often culminates in a pivotal decision-making moment, such as shooting or passing, sees players often resorting more to their NDH. Although future studies could explore these pivotal moments in more detail, this could be influenced by the immediate defensive setup or a player’s positioning or the surrounding situation. All the subjects in this study had prior basketball experience, albeit at different levels. Therefore, it is possible that the defensive format had a stronger influence on the choice of hand used and that the NDHs were used more often than the DHs in the First and in Last phases than the Middle phase.

This study also focused on DC as a one of the dribble phases. Stöckel and Weigelt [7] found that players in higher categories used their NDHs more when dribbling. The main effect of player category was also significant in the two-factor ANOVA of this study. In other words, when all dribbles were analyzed, players in the higher category used both hands equally, while those in the lower category used their DHs more. On the other hand, there was no main effect of category in the one-factor ANOVA for the DC phase in this study, and there was no significant differences in all categories in the one-sample *t*-tests. Therefore, the results of the DC analysis also support the study of Giovanini et al. [6], who raised the possibility that the type of phase affects hand selection. Giovanini et al. [6] also raised the possibility that the frequency of NDH and DH usage might be influenced by different situational factors. The results of this study provide quantitative evidence that the dribbling phases influence hand selection.

Building on the foundation set by Glazier et al. [10], which emphasizes the interaction between the individual, the environment, and the task, our investigation delved into how these interactions influence hand choice during different phases of basketball dribbling. Stöckel and Weigelt [7] suggest that the individual factor, which includes a player’s skill level, mainly determines his or her preference for hand choice during dribbling. Previous studies [6,9,18,19] have highlighted how external factors such as opponent pressure and defensive positioning, which are key aspects of the environment and task, play a central role in influencing technical decisions and play selection in basketball. Our findings, which were conducted in real game settings for each dribbling phase, i.e., with varying environmental and task conditions [10], add to the validity of these findings. Not only are they consistent with the view that individual attributes strongly influence hand choice, but they also support and extend the conclusions of the study by Stöckel and Weigelt [7], suggesting that the choice of the dribbling hand in a basketball game is indeed shaped by a mixture of intrinsic attributes and the specific dribbling phase within which a player is in.

### 4.3. Implications

Our findings may offer insights that could be of interest to basketball coaches. Stöckel and Carey [18] suggested that practitioners and researchers should try to develop practice regimes that allow strongly lateralized players to overcome their inborn hand preference, at least for basketball-specific skills. Therefore, coaches should emphasize the importance of NDH training, especially for younger and less experienced players. Understanding the nuanced effects of dribbling phases can empower trainers to design tailored drills that foster NDH proficiency. By incorporating NDH training into practice, players could potentially improve their dribbling skills and increase their chances of success on the court.

Skill acquisition in sports typically follows a trajectory where players are introduced to skills in simpler conditions, and gradually progresses to more complex scenarios as their proficiency improves. In designing training drills, it is necessary to determine the suitable complexity level of the exercises and tasks to match the players’ proficiency; however, the trainer should know that the setting of a practice session, such as the number of players [19] and defensive pressure [20,21], can affect how a player dribbles. Using our findings, coaches can design training drills that replicate game situations, thus promoting NDH usage. Previously, some studies [3,5] presented methods to facilitate learning on players’ non-dominant sides. The methods presented in these studies are methods that are not used in competitive situations. However, as shown in this study, dribbling phases in a game influence the choice of hand which players’ use. Therefore, deliberately increasing the amount of practice in dribbling phases that include more practice in the First and Last phases would increase players’ dribbling skills with their NDH more than the Middle phase. In addition, competitive phases that encourage DCs, which reduce the tendency to use one side more than the other, should also promote the improvement of the NDH’s dribbling skills. Furthermore, based on the results of this study and the study by Esteves et al. [9], it is possible that the defensive format influences the choice of hand used for dribbling in a game. Therefore, instructing the defense to intentionally protect the DH side would also increase the chance of using the NDH effectively. This would help improve the NDH’s dribbling skills. Thus, focusing on the First, Last, and DC phases when training NDHs could enhance players’ versatility and adaptability on the court more than general training without specific phase settings.

### 4.4. Limitation and Future Directions

The subjects of this study were limited to guards, who generally use the dribble more frequently and require more dribbling skills. Stöckel and Weigelt [7] concluded that there was no difference in the percentage of DH and NDH use by position; however, the results may be different when dribbling phases are divided into different scenes, as in this present study.

This study showed that the basketball-specific skill level as well as the dribbling phase influences hand selection. We have selected the First, Middle, Last, and DC phases as the dribbling phases for analysis. Further detailed analysis of phase classification is needed in the future. As mentioned above, it is expected that factors other than the environment and the task [10] during a dribble, such as the intensity of a defender’s pressure and the direction of the body, might influence the choice of the dribbling hand. Future analysis of dribbling situations based on a defender’s pressure and direction in game situations may provide new insights.

One limitation of this study was the incomplete capture of all dribbles from the available video sources. Although our primary focus was on the dribbling ratio, a comprehensive analysis (especially regarding absolute dribble counts) mitigating this limitation would necessitate videos that document every dribble. Future research aiming to assess both the ratio and absolute dribble counts should prioritize obtaining more exhaustive video data.

While this research provides valuable insights into hand selection during dribbling, it is important to acknowledge the inherent complexities of real-game situations. The dynamics of a live game, including player interactions, strategies, and immediate decisions, among others, can introduce variables that are challenging to fully capture in a study. Our approach, while detailed, offers a simplified perspective of a very intricate and multifaceted gameplay element. Recognizing this, our analysis serves as a foundation, and further studies are encouraged to delve deeper into the nuances of in-game dribbling scenarios.

Furthermore, it would be desirable to examine longitudinal changes in the percentage of NDHs used by specific players and to clarify how the frequency of NDH use increases when a specialized training program to improve NDH skills is applied.

## 5. Conclusions

In conclusion, our study sheds light on how NDH usage in basketball dribbling varies across different player categories and dribbling phases. Overall, our findings are consistent with and support previous research [7], indicating that players at more advanced skill levels tend to favor their NDH more frequently in dribbling. The results indicate that NBA players and college players use their NDHs more frequently than high school players, suggesting that higher skill levels are associated with increased NDH usage. These findings support the significance of NDH dribbling training, particularly for younger and less experienced players, in improving their dribbling skills and overall performance on the court. Furthermore, we observed that the choice of hand during dribbling is also influenced by the specific dribbling phase in which a player is in. Our analysis revealed that specific dribbling phases, particularly the First and Last phases, influence hand choice more than the Middle phase. This suggests that defensive pressure and decision-making factors may play a role in hand selection during dribbling. These insights offer invaluable guidance for coaches. By crafting training drills centered around the First, Last, and DC phases, they can encourage players to use their NDH more frequently. Our study underscores the importance of emphasizing NDH training in basketball coaching programs and provides practical implications for enhancing players’ dribbling abilities. By incorporating NDH training into practice sessions, coaches can help players improve their dribbling skills, increase their chances of success, and enhance their overall performance in competitive settings. Further research is needed to explore the impact of specific situational factors, such as defender pressure and body orientation, on hand selection during dribbling. Longitudinal assessments and specialized training programs can provide deeper insights into the relationship between NDH usage and skill development. Overall, our findings contribute to the existing knowledge on basketball skill acquisition and offer practical guidance for coaches and practitioners aiming to improve players’ dribbling abilities.

## Data Availability

The data presented in this study are available upon reasonable request from the corresponding author.
